# Preparation of highly wettable coatings on Ti–6Al–4V ELI alloy for traumatological implants using micro-arc oxidation in an alkaline electrolyte

**DOI:** 10.1038/s41598-020-76448-w

**Published:** 2020-11-13

**Authors:** Roman Gabor, Martina Doubkova, Simona Gorosova, Karel Malanik, Marta Vandrovcova, Ladislav Cvrcek, Klara Drobikova, Katerina Mamulova Kutlakova, Lucie Bacakova

**Affiliations:** 1 Nanotechnology Centre, VSB – Technical University of Ostrava, 17. Listopadu 15/2172, 70833 Ostrava, Czech Republic; 2 Institute of Physiology of the Czech Academy of Sciences, v.v.i., Videnska 1083, 142 20 Prague 4, Czech Republic; 3 Charles University, Second Faculty of Medicine, V Uvalu 84, 150 06 Prague 5, Czech Republic; 4 VUHZ, a.s., 739 51 Dobra, Czech Republic; 5 Department of Materials Engineering, Faculty of Mechanical Engineering, Czech Technical University in Prague, Karlovo Namesti 293/13, 120 00 Prague 2, Czech Republic

**Keywords:** Biomaterials - cells, Implants, Tissue engineering

## Abstract

Pulsed micro-arc oxidation (MAO) in a strongly alkaline electrolyte (pH > 13), consisting of Na_2_SiO_3_⋅9H_2_O and NaOH, was used to form a thin porous oxide coating consisting of two layers differing in chemical and phase composition. The unique procedure, combining MAO and removal of the outer layer by blasting, enables to prepare a coating suitable for application in temporary traumatological implants. A bilayer formed in an alkaline electrolyte environment during the application of MAO enables the formation of a wear-resistant layer with silicon incorporated in the oxide phase. Following the removal of the outer rutile-containing porous layer, the required coating properties for traumatological applications were determined. The prepared surfaces were characterized by scanning electron microscopy, X-ray diffraction patterns, X-ray photoelectron spectroscopy, atomic force microscopy and contact angle measurements. Cytocompatibility was evaluated using human osteoblast-like Saos-2 cells. The newly-developed surface modifications of Ti–6Al–4V ELI alloy performed satisfactorily in all cellular tests in comparison with MAO-untreated alloy and standard tissue culture plastic. High cell viability was supported, but the modifications allowed only relatively slow cell proliferation, and showed only moderate osseointegration potential without significant support for matrix mineralization. Materials with these properties are promising for utilization in temporary traumatological implants.

## Introduction

Titanium and titanium alloys are materials with an increasing share of applications in many fields, primarily in the aerospace industry, in healthcare, in the automotive industry, and now also in the offshore industry. Because of its favorable mechanical properties, Ti–6Al–4V ELI alloy is currently one of the most widely-used titanium alloys for medical applications. The applications are successfully realized despite the presence of aluminum and vanadium, which are potentially harmful alloying elements that might be released in the form of ions from the bulk material under specific tribocorrosion conditions^1,2^. However, the presence of these alpha and beta stabilizing elements (Al and V, respectively) provides the alloy with great corrosion resistance and with suitable mechanical properties, such as moderate tensile and fatigue strength, formability and good creep resistance^3,4^.

The basic requirement for materials used in biomedical implants is that they should be biocompatible. This involves mutual interplay among a number of key material properties that define the best-possible contact with an internal environment within the human body. Not only the surface morphology and the physical properties of the material are important, but also the chemistry of the surface layer and the physiological environment to which the implants are to be exposed. By selecting a suitable modification method, it is possible to achieve a functional surface that allows for desired biological interactions between a medical implant and the tissue, depending on its intended application. For these purposes, various mechanical methods (fine working, grinding, tumbling, blasting), chemical methods (pickling, CVD, anodic oxidation, sol–gel) and physical methods (PVD, thermal spraying, ion implantation) have been investigated and are now in use in practical applications^5^.

In recent years, the most widespread surface modification method for titanium and its alloys has been electrochemical anodization, in combination with mechanical and chemical methods for the necessary surface pre-treatment. Mechanical methods produce a specific surface topography and roughness, remove surface contamination and improve surface adhesion^6^. Degreasing and pickling are used as chemical pre-treatments to remove contaminants and the thin naturally occurring oxide layer (< 10 nm)^7^.

A promising technique which has emerged in recent years is micro-arc oxidation (MAO), also known as plasma electrolytic oxidation. MAO technology is used to prepare a very thin porous oxide layer with variable properties. This layer markedly improves the basic features of titanium alloy by decreasing the risk of potentially harmful ion release from the bulk material^8^, and by providing enhanced corrosion resistance of the material^9^. These properties of the materials then have a considerable impact on the interaction between a metallic implant and the surrounding cells^5^. In addition, the MAO method is relatively inexpensive and is non-toxic to the environment^10^.

The MAO method is also known in the literature as Anodische Oxidation unter Funkenentladung (ASD), as plasma electrolytic oxidation (PEO), and as the anodic plasma-chemical process (APC). This material surface modification method is also clinically used under commercial names, e.g. Ticer (ZL-Microdent Breckerfeld, Germany), TiUnite (Nobel Biocare Holding AB, Switzerland) or Osstem (Osstem Implant Co., Korea). Other similar coating methods available for clinical use in orthopedics include DOTIZE (DOT GmbH, Germany) and the TioDark process (KKS Ultraschall AG, Switzerland)^11^.

MAO can be performed in an acidic or an alkaline electrolyte using the galvanostatic or potentiostatic mode of operation^12–14^. In order to achieve the required tribological, chemical, structural and biocompatible properties of the implant surface by electrochemical anodization, it is necessary to optimize several parameters of the process, e.g. the time, the voltage, the current density, the electrolyte composition and the temperature^15^. An important criterion for achieving the desired electrolyte effect is the presence of additives in alkaline or acidic electrolytes. The presence of additives such as calcium acetate hydrate (C_4_H_6_O_4_Ca∙H_2_O), disodium hydrogen phosphate (Na_2_HPO_4_), sodium silicate and Na_2_SiO_3_∙9H_2_O in the alkaline electrolyte allows the chemical species to be incorporated into the coating. These species then influence its thickness, roughness, corrosion resistance, tribology, and also the adhesion and proliferation of cells on the coating^16,17^. Silicate coatings prepared by MAO show improved tribological properties, corrosion resistance^18^, and they can also modulate the bone growth^19^.

In the field of traumatology, it is important to select a suitable electrolyte to achieve the desired chemical composition for the cell–metal interaction in the final application. A combination of specific process conditions is used to ensure that the plasma discharge develops at the desired layer thickness. These process conditions and further treatment are used to ensure the unification of the surface, along with the desired topography, phase composition and surface wettability.

The Ti–6Al–4V ELI samples used in this study were modified in alkaline electrolytes with the use of MAO technology equipped with a unipolar pulse source. The aim was to prepare a coating suitable for temporary traumatological implants, e.g. screws, nails, wires, staples or splints. Our expectation for this coating was that it should be biocompatible, non-cytotoxic and supportive for cell viability. At the same time, however, it should not promote firm osseointegration, which would hamper the removal of a temporary implant from the body. A novel technological procedure is proposed in this study aimed at eliminating the disadvantages of MAO, which are linked to the high surface roughness of the initially deposited oxide layer^20^. The results showed that two layers with a different phase composition were prepared during the MAO process. The outer porous layer was then removed in order to reduce the average roughness and the TiO_2_ phase (rutile). The phase composition of the newly-developed coating correlated well with the choice of the electrolyte. It showed dependency on the total energy of each pulse, which was ensured using the unipolar source.

The interactions of cells with the modified surface of the samples in this study were investigated *in vitro,* using human osteoblast-like cells of the Saos-2 line. The suitability of the surface properties of the samples and their effect on the cell behavior were evaluated at various stages of the cell culture. The following signs of the cell-material interactions were evaluated: the number, the spreading and the morphology of the initially adhering cells, the cell population density in the subsequent time intervals, which is an indicator of cell proliferation, the cell viability, which is an indicator of potential material cytotoxicity, the collagen type I deposition, the gene expression of selected osteogenic markers (collagen type I, alkaline phosphatase and osteocalcin), and calcium deposition, which is a sign of bone matrix mineralization. The cell behavior was then correlated with the physicochemical properties of the material surface, i.e. its topography, roughness, wettability and the chemical composition of the surface layer. The results were also compared with those obtained in cells cultured on the control samples of MAO-untreated alloy (Ctrl) and on standard polystyrene cell culture plates (PS).

## Results and discussion

### Morphology of the MAO-coated Ti–6Al–4V surface

**Figure 1:**
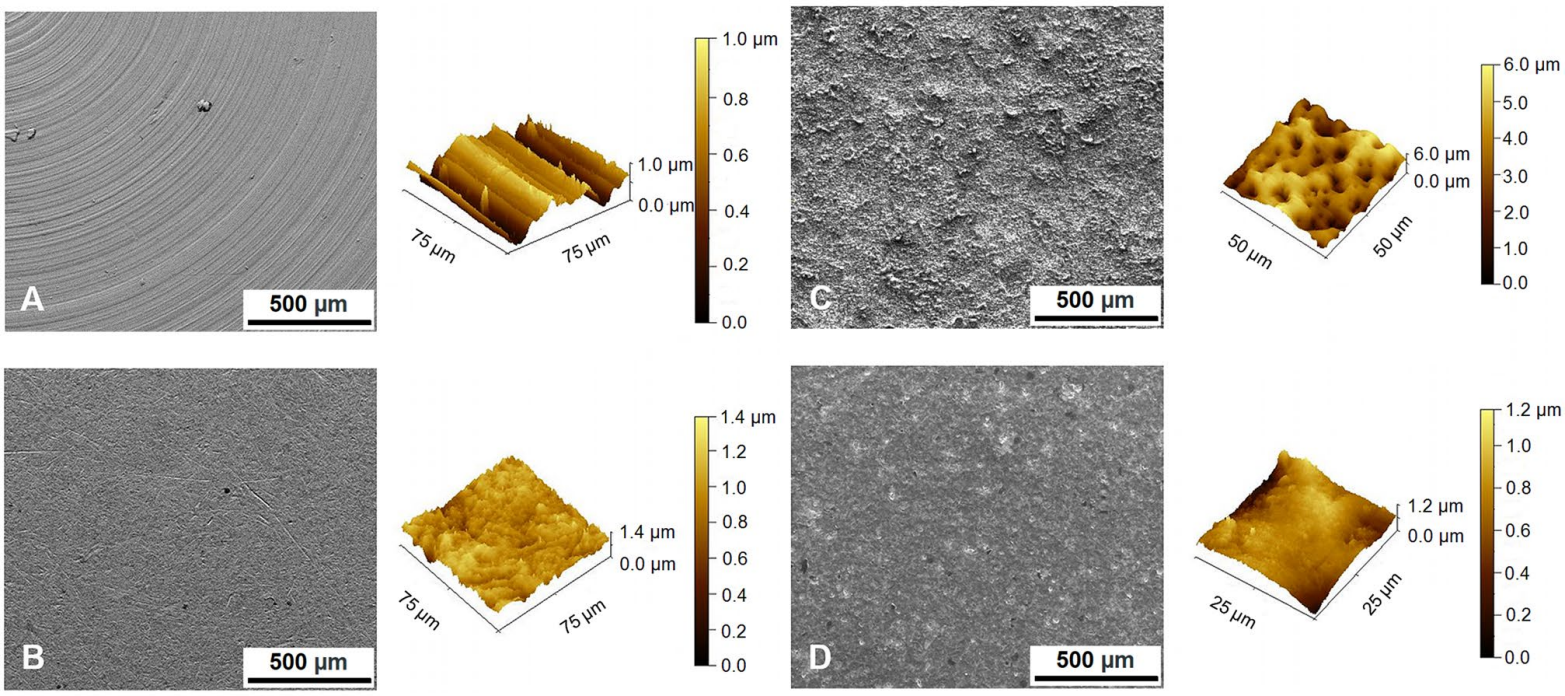
Surface morphology of samples before the MAO process (**A**, **B**) and after the MAO process (**C**, **D**). (**A**) A sample after chip machining; (**B**) a sample after mass finishing by the vibration tumbling technology, which served as a control sample (Ctrl); (**C**) a sample after the MAO process (sample MA01); (**D**) a sample after blasting (sample MA01-blasting). Left images: FEI FE-SEM Quanta 450 FEG microscope, bar: 500 μm. Right images: AFM, Solver NEXT (Gwyddion 2.56 software, https://gwyddion.net), bars: 1.0 μm, 1.4 μm, 6.0 μm and 1.2 μm, respectively.

The surface roughness of the tested materials was evaluated by measuring the parameters R_a_ (average roughness), R_z_ (maximum height of the profile) and RS_m_ (mean spacing of the profile irregularities). The surfaces of the samples after chip machining had roughness R_a_ = 0.65 ± 0.02 µm and R_z_ = 3.42 ± 0.15 µm. Surfaces with inlet roughness R_a_ = 0.28 ± 0.01 µm, R_z_ = 1.88 ± 0.05 µm and RS_m_ = 340 ± 0.03 µm were achieved using vibration tumbling technology (Table 1, Fig. 1) and were used as control samples (Ctrl).

**Table 1:** Characterization of samples: final surface roughness of samples, static contact angle with liquids, solid surface free energy, comparison of coefficients of friction and widths of tracks in air and in phosphate-buffered saline (mean values ± SD). Ctrl: MAO-untreated Ti–6Al–4V samples; MA01: samples treated with MAO; MA01-blasting: samples treated with MAO with the outer porous layer removed by blasting; PS: cell culture polystyrene.

Parameter/sample	Ctrl	MA01	MA01-blasting	PS
**Roughness (µm)**				
R_a_	0.28 ± 0.01	1.50 ± 0.04	0.50 ± 0.02	N/A
R_z_	1.88 ± 0.00	6.49 ± 0.25	2.57 ± 0.03	N/A
RS_m_	340.00 ± 0.03	62.10 ± 0.01	127.10 ± 0.01	N/A
**Contact angle (°)**				
H_2_O	71.8 ± 5.8	15.6 ± 4.6	35.4 ± 9.3	76.5 ± 1.6
Glycerol	63.3 ± 3.7	17.8 ± 4.2	30.4 ± 6.7	71.2 ± 1.2
**Solid surface energy (mN/m)**				
Total	33.4 ± 18.84	70.9 ± 6.98	60.0 ± 24.69	28.3 ± 5.23
Dispersive component	18.1 ± 9.67	14.8 ± 2.54	17.8 ± 9.60	12.5 ± 2.48
Polar component	15.3 ± 9.17	56.1 ± 4.45	42.1 ± 15.08	15.8 ± 2.76
**Coefficient of friction µ**				
Air	0.68	0.63	0.64	N/A
PBS	0.43	0.39	0.72	N/A
**Track width (mm)**				
Air	0.68 ± 0.05	0.22 ± 0.01	0.12 ± 0.01	N/A
PBS	0.48 ± 0.01	0.23 ± 0.01	0.16 ± 0.01	N/A

**Figure 2:**
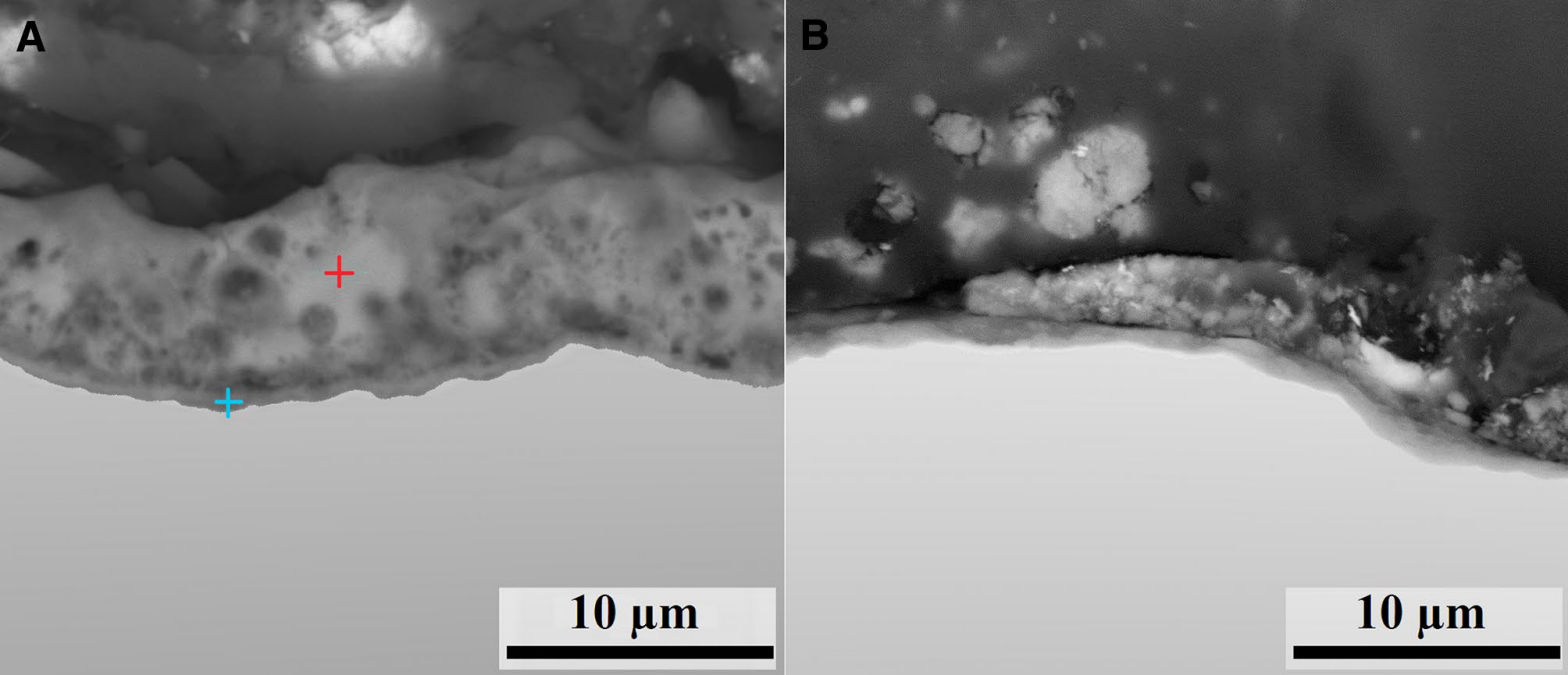
(**A**) Cross sections of the layer coating produced after the MAO process (sample MA01; magnification 5000 ×). The cross mark indicates the representative location for a chemical analysis of the outer layer (red label) and the inner layer (blue label). (**B**) Cross section depicting the layer thickness after the MAO process and blasting (sample MA01-blasting). FEI FE-SEM Quanta 450 FEG microscope (APEX Software for EDX 2.0, www.edax.com), bar: 10 μm.

From the determined dependence of the surface roughness parameters R_a_ and R_z_ on the mechanical pre-treatment and electrochemical anodization procedures (Fig. 1), it is apparent that the surface roughness increases during the anodic oxidation process. The surface roughness parameters of the samples after the MAO procedure, referred to as MA01 samples, were R_a_ = 1.50 ± 0.04 µm and R_z_ = 6.49 ± 0.25 µm. The mean spacing of the irregularities, described by the RS_m_ parameter, was reduced from the original 340 ± 0.03 to 62.10 ± 0.01 µm (Table 1). A uniform inner layer and a highly porous outer layer were formed on the sample during the MAO process (Fig. 2). Its chemical composition was determined from metallography cross sections of the layer, revealing the different silicon contents in individual layers. While the content of Si was relatively high in the outer porous oxide layer, the inner layer formed during the MAO process contained several times less Si than the outer layer (Table 2). The outer porous oxide layer reached a thickness of 6.86 ± 1.03 µm, whereas the thickness of the inner oxide layer was only 0.83 ± 0.11 µm. However, parameters such as these could significantly affect the tribological and biological properties of the surface, and its potential for application in the field of traumatology. It was therefore necessary to adjust the roughness of the anodized surface with respect to its desired final application for biomedical implants. In order to achieve more suitable surface roughness parameters, the outer porous layer was mechanically removed by blasting. During blasting, the porous outer layer of the MA01-blasting sample was removed, while the thickness of the inner layer remained the same due to its homogeneity and hardness. After this treatment, the final surface roughness parameters of the MA01 sample treated by blasting (referred to as MA01-blasting samples) decreased to Ra = 0.50 ± 0.02 µm and Rz = 2.57 ± 0.03 µm, and the mean spacing of the irregularities increased to 127.10 ± 0.01 µm (Table 1). Therefore, the blasting treatment decreased the surface roughness of the MA01 samples.

**Table 2:** Chemical composition of the surface layers.

Element	Inner layer		Outer layer	
	wt.%	at.%	wt.%	at.%
O	32.6	55.7	52.4	66.4
Na	2.2	2.6	1.9	2.2
Al	3.7	3.8	–	–
Si	7.1	6.9	41.5	29.7
Ti	52.4	29.9	3.9	1.5
V	1.8	0.9	0.2	0.1

The increase in the surface roughness of the MAO-treated samples is dependent on the input energy increase and a stronger spark discharge inside the channels. With increasing voltage and current density, the material melts and immediately oxidizes at the metal–electrolyte interface inside the channels^21^. The formation of an inner layer (the so-called passive layer) 0.83 ± 0.11 µm in thickness was therefore related to the linearization of the increase in the voltage input. High voltage is required to allow the formation of a spark discharge, accompanied by an isolated discharge, which can be observed on the surface of the material. The strong discharge with a subsequent current drop resulted in the growth of a dielectric porous outer layer 6.86 ± 1.03 µm in thickness^22^. According to Zhang et al.^23^, during the formation of the outer porous layer a much lower voltage drop occurs than when the inner layer is forming. This can be explained by the much lower resistance of the outer porous layer in comparison with the compact inner layer. This corresponds with the slow growth of the outer porous layer after reaching spark discharge.

### XRD characterization

**Figure 3:**
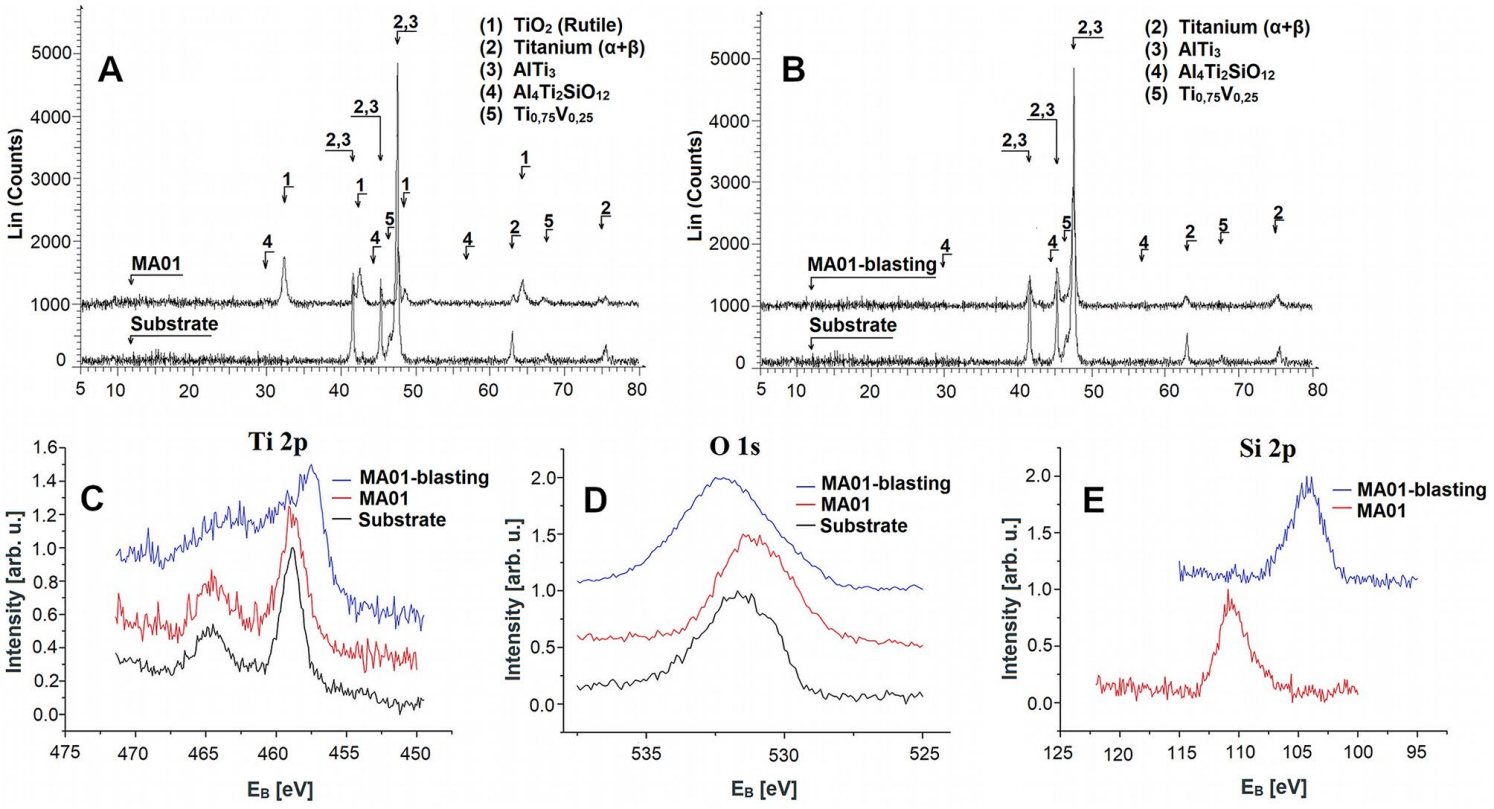
XRD patterns and XPS spectra of the substrate (Ctrl sample), MA01 and MA01-blasting: (**A**) XRD patterns after MAO coating (sample MA01), (**B**) XRD patterns after removal of the outer layer by blasting (sample MA01-blasting), (**C**) XPS spectrum Ti 2p, (**D**) XPS spectrum O 1s, (**E**) XPS spectrum Si 2p. Bruker D8 Advance diffractometer (DIFFRAC.SUITE EVA V3—XRD Software, www.bruker.com), XPS (X-ray Photoelectron Spectroscopy) and Omicron EA-125 electron energy analyser and a dual anode X-ray source (XPS Lab, www.scientaomicron.com).

The diffraction records of the samples before the application of MAO, i.e. after mechanical treatment, of samples after MAO and of samples after MAO with the porous layer removed by shot blasting (Fig. 3) all confirm compliance with the presumed presence of rutile and silicate phases. The following crystalline phases were confirmed in the samples: (1) TiO_2_ oxidic phase in the crystalline modification of rutile, (2) titanium (α + β), (3) AlTi_3_, (4) Al_4_Ti_2_SiO_12_, and (5) Ti_0,75_V_0,25_. The results of the diffraction records show that the rutile phase that was present was removed by blasting the outer porous surface in the sample of MA01-blasting type. According to the results of EDX and XRD analyses and the phases that were shown to be present, we can conclude that there is significant enrichment of the outer porous layer with silicon. This was confirmed in the Al_4_Ti_2_SiO_12_ crystalline phase that was present. According to Wang et al.^24^, we can also assume the presence of amorphous SiO_2_, which was incorporated into the coating from the electrolyte solution.

The results of the XPS analysis (Fig. 3) confirmed the presence of a passivation layer on the substrate. The composition of the coatings was revealed using the EDX and XRD methods. Major elements that were identified were Ti, O and Si. The binding energy of Ti 2p_3/2_ in the spectrum of the substrate sample and MA01 was determined, with the peak at 458.8 eV (Fig. 3C) corresponding to the presence of TiO_2_ (rutile)^25^. The shape of the spectrum and the shift to binding energy of 457.4 eV in the MA01-blasting sample (Fig. 3C) may correspond to a change in the structure following surface blasting and removal of the outer layer. This value does not correspond to any of the tabulated values of the binding energies and chemical states of the elements. The value probably corresponds to the presence of an imperfectly removed inhomogeneous outer layer formed mainly of the TiO_2_ phase (rutile). This phase was not confirmed in the inner layer using the XRD method, due to the different sensitivities of the two methods.

The present phases and the changes in the structure on the surface following the application of MAO and blasting of the outer layer affected the resulting shifts of the O 1s peaks to higher binding energy values of 532.3 eV (Fig. 3D). The chemical presence of Ti in the form of TiO_2_ was confirmed from the peak energy O 1 s 530.7 eV (Fig. 3D). The resulting Si 2p spectrum of the MA01-blasting sample (Fig. 3E) confirms—based on the binding energy value of 104.1 eV—the presence of SiO_2_, in correlation with the peak in the O 1s spectrum located around the value of 532.3 eV^26^ According to Muhaffel et al.^27^, the development of amorphous SiO_2_ in MAO coatings occurs in connection with a low cooling rate. The visible shift of the peak of the Si 2p MA01 spectrum may be related to the inhomogeneity of the coating and to the presence of SiO_2_ grains (insulator), which may cause different charging of individual components of the coating.

### Surface wettability

Contact angle measurements revealed that MAO treatment significantly increased the hydrophilicity of the Ti–6Al–4V samples in comparison with the MAO-untreated controls (Ctrl). The contact angle of both water droplets and glycerol droplets on the MA01 sample was several times lower than on the Ctrl and MA01-blasting samples. With a water contact angle of 16°, the wettability of MA01 was therefore the highest of all samples. Similarly, the polar component of the surface energy of MA01 was also higher than in the Ctrl and in the MA01-blasting samples (56 mN/m, in comparison with 15 mN/m and 42 mN/m, respectively). Additional surface treatment of the samples by shot blasting, however, changed the surface roughness of the samples and thus reduced their wettability significantly. This change is demonstrated by a higher contact angle (35°) and a lower polar component of the surface energy (42 mM/m) of the MA01-blasting sample. The reference Ctrl and PS samples (contact angle around 70°) can be regarded as moderately hydrophilic, which is considered beneficial for cell adhesion and growth. Mean values of the contact angles and the surface energy are given in Table 1.

The wettability of a material is particularly important, as it can influence the adsorption of the proteins supporting cell adhesion and their spatial organization^28–30^. Advantageous material properties are reflected in the cytoskeleton and in the cell morphology, e.g. the cell being more polygonal in shape and occupying a larger area^29,31,32^. However, wettability is a result of combined properties of the material surface, i.e. topography, chemical composition and surface charge, all of which interact to affect the cell behavior. It is therefore difficult to assess these parameters individually.

### Tribological characteristics

**Figure 4:**
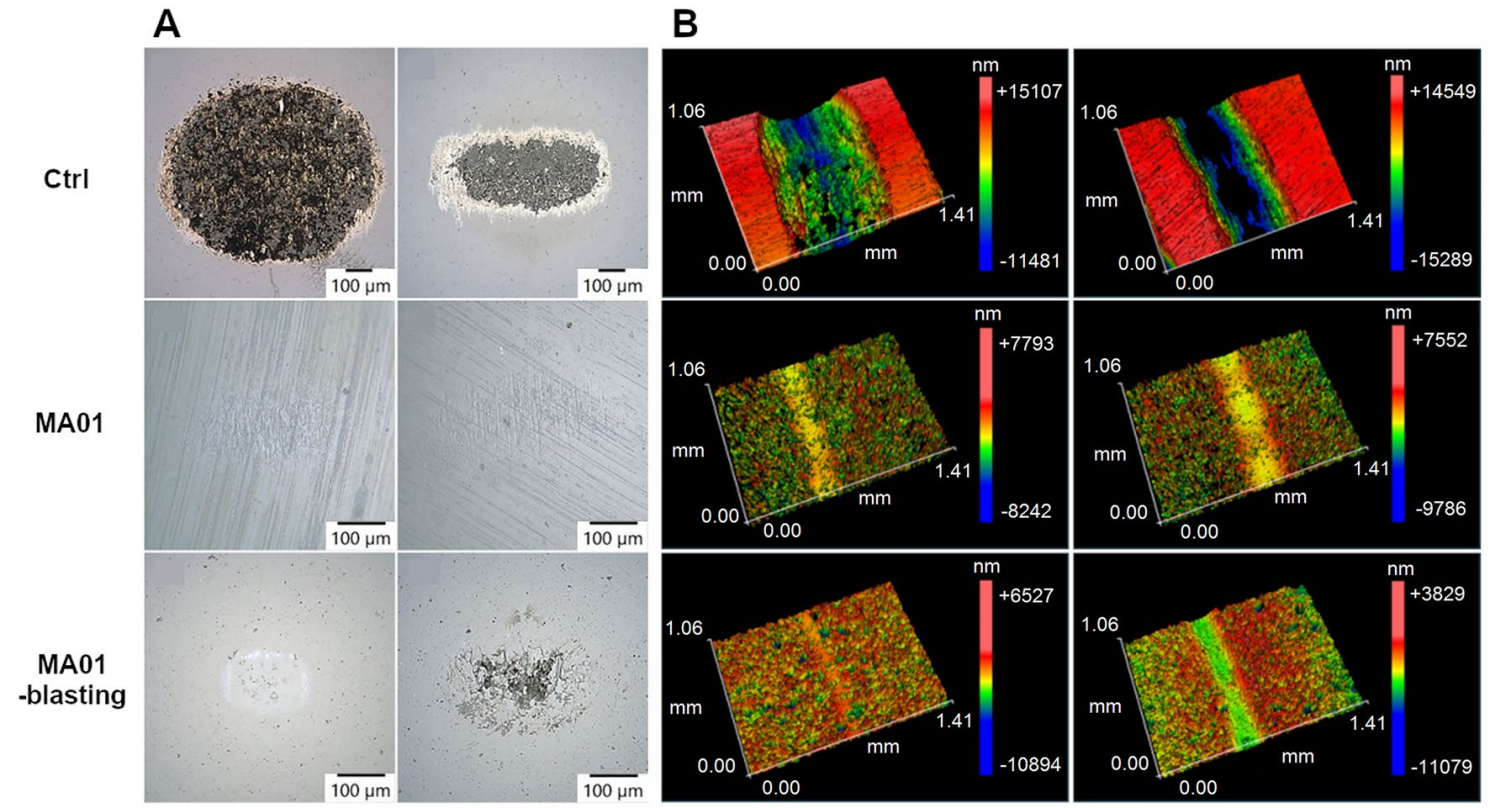
(**A**) Wear of the Al_2_O_3_ ball in the air (left) and in the PBS (right). (**B**) Profile of the wear track in the air (left) and in the PBS (right). Olympus DSX1000 digital microscope and Zygo NewView 72003D optical profilometer (Mx Software 7.0, www.zygo.com).

The average values of the coefficients of friction tested by a pin-on-disc tribometer were in the range of 0.63–0.68 in air. In a phosphate-buffered saline (PBS) solution, which acted as a lubricant, the values decreased to the range of 0.39–0.43. However, this was not the case for the MA01-blasting sample, in which the coefficient of friction increased to 0.72. In this case, the ball surface was damaged by adhesion wear in PBS and by abrasion in air. For the MA01 samples, the ball wear was minimal. Only scratches caused by contact with the tops of the rough surface were visible on the surface of the ball. For the Ctrl samples, the counterpart material adhered to the surface of the ball (Table 1; Fig. 4A).

The wear was not evaluated by the standard procedure based on the wear rate, because the surface of the samples showed relatively high roughness. The wear track profile then did not correspond to the actual volume loss of the material, as in the case of a compact material. Rather than the wear rate, the width of the wear track was evaluated, which is more indicative of the wear mechanism. The wear track profiles measured by a 3D optical profilometer are shown in Fig. 4B and the track widths are shown in Table 1. The track widths confirmed a significant decrease in wear compared to the Ctrl samples. The lowest wear was obtained with the MA01-blasting sample, but its lower initial surface roughness must be considered.

### Adhesion, growth and differentiation of Saos-2 cells on samples

**Figure 5:**
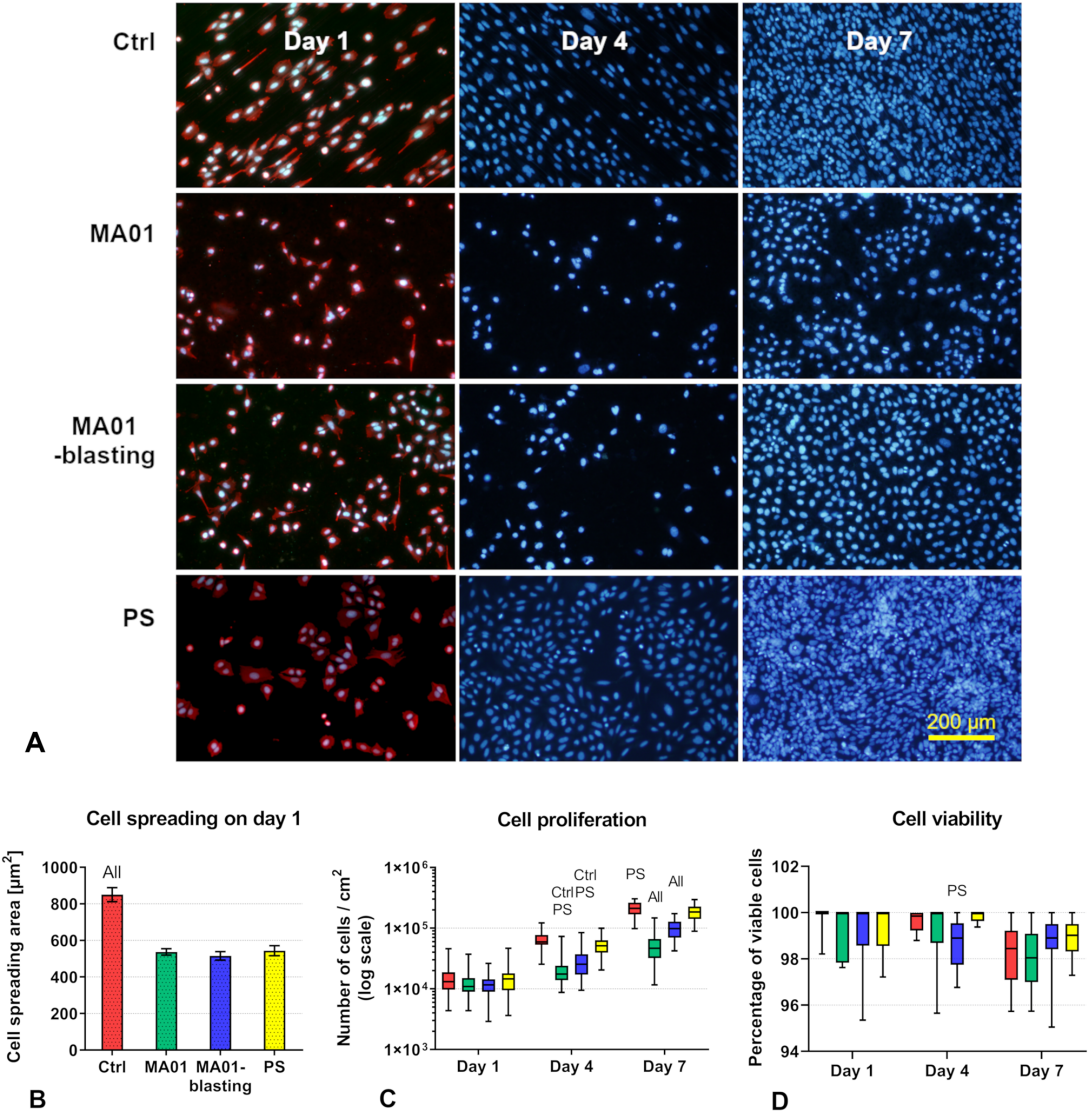
(**A**) Morphology and proliferation of cells growing for 1, 4 and 7 days on the samples (columns two to four): Olympus IX 51 microscope (obj. 10 ×), bar: 200 μm. The cells were stained with fluorescent dyes Texas Red C_2_-maleimide (red; stains cytoplasm proteins) and/or Hoechst #33258 (blue; stains cell nuclei). (**B**) Cell spreading areas on samples. Mean ± S.E.M. from 150 to 217 measurements per sample. (**C**) Cell proliferation dynamics and (**D**) Viability of cells on samples. Cells were cultivated on the samples for 1, 4 and 7 days in NORM medium. Data from 10 measurements for each sample in quadruplicate. The box plot bold black central line shows the median; its outer edges represent the 1st and 3rd quartile (Q1 is the 25th percentile, Q3 is the 75th percentile of the sample), the whiskers depict the maximum and minimum values. Kruskal Wallis One Way ANOVA on Ranks, Dunn’s test; the statistical significance (*p* ≤ 0.05) in comparison with specific samples is marked above individual columns (“All” indicates statistical significance vs. all other samples).

On day 1, the cell morphology on the Ctrl, MA01 and MA01-blasting samples was similar, consisting mainly of elongated spindle-shaped cells with long protrusions, in contrast to PS, where the cells assumed more polygonal shapes and spread over larger areas of the sample (Fig. 5A). These differences in cell shape can be explained by the irregularities on the Ti–6Al–4V-based samples, which were created by machining, by vibration tumbling and by MAO treatment. It is clearly visible that the cells on the Ctrl samples are aligned in parallel with the grooves and ridges created by the mechanical treatment (Fig. 5A). In a study performed on fibroblasts and osteoblasts cultured on ground titanium surfaces, the oriented cells had a higher density of focal contacts, and showed better organization of the cytoskeleton and stronger actin fibers than randomly distributed cells^33^. The initial cell adhesion, evaluated after 1 day of culture, showed that cells adhered to the control Ti–6Al–4V samples (Ctrl) with significantly larger spreading areas than to the MA01 and MA01-blasting samples, and even than to the standard cell culture polystyrene wells (PS). At the same time, the sizes of the cell spreading areas on the MA01, MA01-blasted and PS samples were similar, and without statistically significant differences (Fig. 5B). The cell population densities on the MA01 and MA01-blasting samples were slightly lower than on the Ctrl and reference PS samples. However, these differences were not statistically significant after 1 day of cultivation (Fig. 5C).

On day 4 after seeding, the cells on the MA01 and MA01-blasting samples grew in a more scattered pattern, where individual cells were usually separated each from other (Fig. 5A). The cell population densities on these samples were significantly lower than on the Ctrl and PS samples, which indicated a slower proliferation rate of the cells on both types of MAO-modified samples (Fig. 5C). This slower proliferation rate was evidently more pronounced on the MA01 samples. The cells on these samples were not able to reach confluence before the end of the experiment, i.e. up to day 7, when the cells on the MA01-blasting samples were subconfluent, and the cells on the Ctrl and particularly on the PS samples had reached full confluence (Fig. 5A). On day 7, the MA01 samples exhibited the lowest cell population density, which was significantly lower than on the MA01-blasting samples. At the same time, the cell population densities on both the MA01 and the MA01-blasting samples were significantly lower than on the Ctrl and PS samples, and the cell population density on the Ctrl samples was slightly but significantly higher than on the PS samples (Fig. 5C).

In spite of a smaller spreading area and a slower proliferation rate, the cells on the MA01 and MA01-blasting samples retained their spindle-shaped morphology, which was similar to the morphology of the cells on the Ctrl alloy samples. This can be considered as a sign of good viability of the cells on both types of MAO-modified samples. In accordance with this finding, the percentage of viable cells was generally high, and did not differ considerably among the samples in the assessed time intervals (days 1, 4, and 7). The lowest median values for each time interval were: 100% viability on day 1 (all samples), 98.89% viability on day 4 (MA01-blasting) and 98.04% viability on day 7 (MA01) (Fig. 5D). With the exception of day 4, when the cell viability on MA01-blasting was slightly but significantly decreased in comparison with PS, no significant differences were detected among the samples.

The surfaces of the control Ti–6Al–4V alloy samples (Ctrl) and the standard polystyrene culture wells (PS) exhibited very similar wettability values (due to the contact angle and the polar component of the solid surface energy); it is therefore not surprising that the cell proliferation on these samples was very similar. These surfaces with a water drop contact angle of about 70° can be considered moderately wettable, i.e. suitable for the adhesion, migration and proliferation of cells (for a review, see^29^). It is known that cell adhesion to artificial materials is mediated by extracellular matrix (ECM) proteins, such as fibronectin, vitronectin, collagen or laminin. These proteins are adsorbed on the materials from biological fluids, including the serum supplement of cell culture media. On moderately wettable surfaces, these proteins are adsorbed in a flexible, physiological conformation, where specific amino acid sequences in these proteins, e.g. RGD, are well-accessible for the adhesion receptors on the cells, e.g. integrins. The adhesion receptors are then clustered into focal adhesion plaques, where they communicate with various structural and signaling molecules, and they deliver mechanical and biochemical signals to the cells. These signals then govern the cell behavior, including the proliferation activity of the cells^29,32^. However, on highly hydrophilic surfaces, the adsorption of cell adhesion-mediating proteins is weak and unstable, and these proteins cannot provide an adequately firm anchor for the adhering cells. Although the specific amino acid sequences in the protein molecules are still accessible for cell adhesion receptors, these receptors cannot be sufficiently assembled into focal adhesion plaques and cannot sufficiently support the cell spreading that is a prerequisite for further cell proliferation (for a review, see^29^). This could offer an explanation for the slower proliferation of the cells on the MAO-treated samples, particularly on the MA01 samples, where the water drop contact angle was the lowest, i.e. the hydrophilicity was the highest. A similar phenomenon was observed on highly hydrophilic oxygen-terminated nanostructured diamond surfaces (water drop contact angle lower than 2°), which almost completely resisted the adhesion of human bone marrow mesenchymal stem cells. At the same time, less hydrophilic hydrogen-terminated nanodiamond surfaces (contact angle 86°, i.e. comparable to the contact angle on the Ctrl and PS in our study) provided good support for the adhesion, spreading and growth of these cells^34^. Another example is a poly(DL-lactide) surface tethered with polyethylene oxide (PEO) chains, in which relatively high surface hydrophilicity (water drop contact angle less than 30°) was coupled with high mobility of the PEO chains. This disabled the adsorption of cell adhesion-mediating proteins, and they were therefore non-adhesive for vascular smooth muscle cells^35^.

The surface roughness of the material is another important parameter regulating the adhesion and growth of cells and the osseointegration of the implant. However, it seems that there is no consensus about universal roughness values supporting or hampering cell adhesion and growth. Several studies performed on various cell types and on various materials have shown that cells adhere, grow and differentiate better on rough surfaces than on smooth, polished surfaces. In those studies, the dimensions of the irregularities on the surface of the material were much bigger or much smaller than the bone cells themselves. In other words, the surface roughness in these cases was either in macroscale, or in submicron-scale or even in nanoscale^33,36–39^. Macroscale surface roughness, i.e. roughness distinguishable by the human eye (from at least 100 μm to millimeters or more), is not usually felt by cells which are spread over tens of micrometers, and it usually contributes to better mechanical anchorage of the implant in the bone tissue. Submicron roughness, and particularly nanoroughness, of a material can imitate the physiological irregularities within the ECM, such as various curvatures, helices or side chains in organic molecules, and crystals in the inorganic component of ECM, e.g. in the bone tissue, and usually supports the adhesion and growth of cells. However, irregularities several micrometers in size can hamper the adhesion, the spreading and the subsequent growth of the cells. The cells are forced to adhere in depressions among the prominences, which can limit their spreading area, or they need to bridge the prominences and cannot use the entire cytoplasmic membrane for adhesion. The cells can also adhere on both depressions and prominences, but this often leads to deformations of the cytoplasmic membrane, the actin cytoskeleton and the cell nucleus, and leads to delayed maturation of focal adhesions^38^ (for a review, see^29^). The surface roughness of both MAO-modified samples, particularly the R_z_ parameter, was in the micron-scale, and together with high surface hydrophilicity, it can explain the lower spreading and proliferation rate of Saos-2 cells on these surfaces.

Taken together, our results indicate that high surface wettability and micron-scale surface roughness had a synergistic limiting effect on cell adhesion and growth. This limiting effect was most pronounced in the MA01 samples, i.e. in the samples with the highest hydrophilicity and the highest surface roughness. However, it should be taken into account that the mean spacing of the profile irregularities (RS_m_), which is ~ 62 µm in MA01 and ~ 127 µm in the MA01-blasting samples, seems to be sufficiently long to accommodate the cells. The cells usually need to spread over tens of micrometers for their good functional performance on a material. It can therefore be assumed that the high surface wettability had a predominant effect limiting the spreading and growth of the cells on both MAO-treated samples. In any case, materials supporting high cell viability of bone cells, but not high proliferation activity, are desirable for temporary bone implants, in which firm osseointegration would hamper removal of the implant.

**Figure 6:**
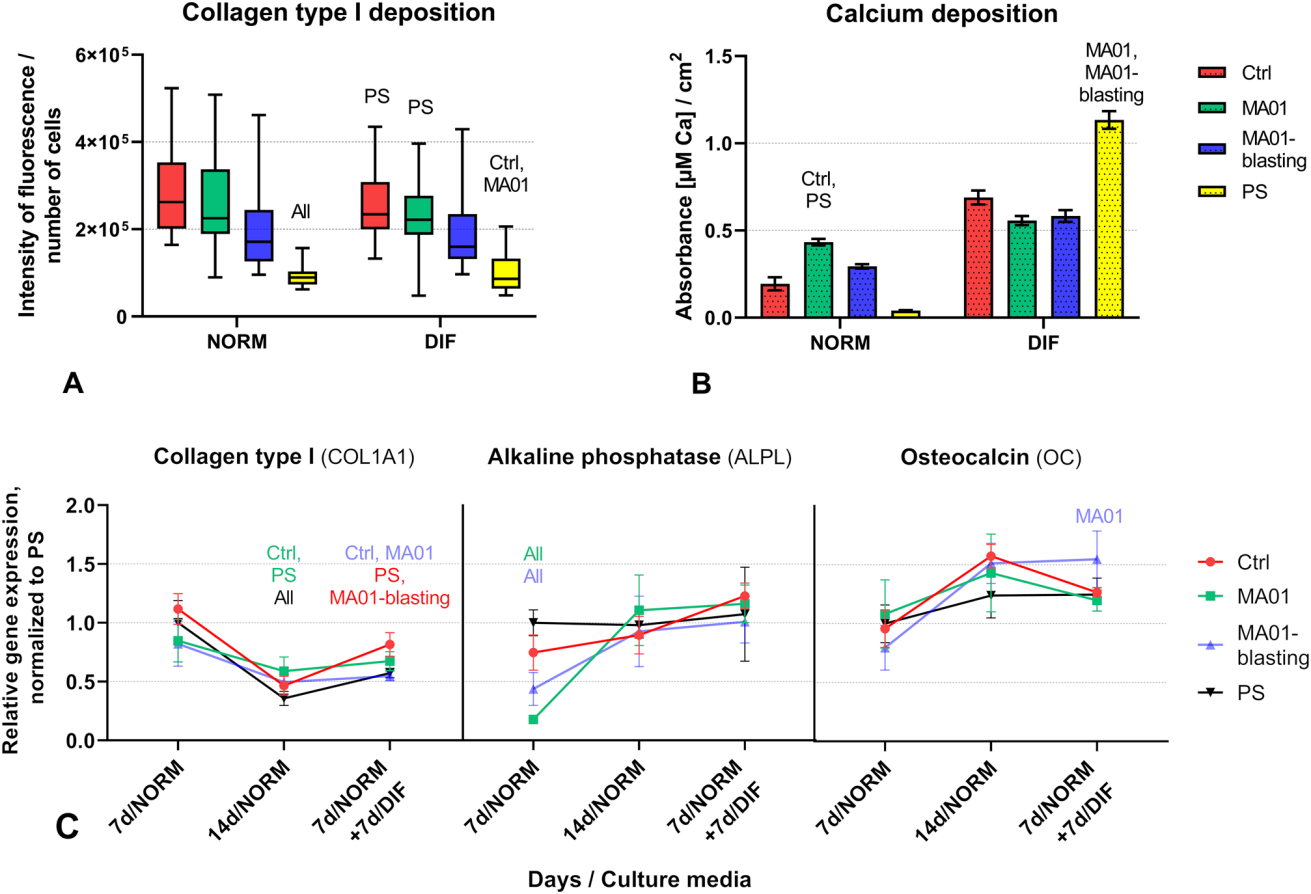
Quantification of collagen type I production (**A**) and calcium deposition (**B**) by cells after 14 days of cultivation either in the NORM medium (stimulating proliferation) or after 7 days in the NORM medium and then 7 days in the DIF medium (stimulating osteogenic differentiation). The box plot bold black central line shows the median value of data from 10 measurements for each sample in triplicate; its outer edges represent the 1st and the 3rd quartile (Q1 is the 25th percentile, Q3 is the 75th percentile of the sample), and the whiskers depict the maximum and minimum values. The bar chart displays mean ± S.E.M. from 2 measurements for each sample in triplicate. Kruskal Wallis One Way ANOVA on Ranks, Dunn’s test; statistical significance (*p* ≤ 0.05) is marked above individual columns (“All” indicates statistical significance vs. all other samples). (**C**) Relative gene expression of cells cultivated for 7 and 14 days in the NORM medium and the expression of cells cultivated for 7 days in the NORM medium and then 7 days in the DIF medium. The graph compares the relative expression (quantified by the 2^−ΔΔCt^ method), expressed in relation to GAPDH, and normalized to the gene expression on PS on day 7 (PS_7d/NORM, calibrator). Mean ± S.D. from 3 measurements on each sample. One Way ANOVA, Holm–Sidak test, statistical significance (*p* ≤ 0.001).

The early osteogenic cell differentiation was estimated by the amount of type I collagen produced by cells cultivated on the tested samples. The cells were grown for 14 days (i.e., for 7 + 7 days) in the standard cell culture medium (NORM), or for the first 7 days in the NORM medium and then for 7 additional days in an osteogenic differentiation-promoting medium (DIF), as previously described. As revealed by immunofluorescence, the cells produced type I collagen in comparable amounts under both types of cultivation conditions (Fig. 6A). At the same time, the cells on the MA01 samples and also on the MA01-blasting samples produced amounts of type I collagen comparable with those on the MAO-untreated alloy samples (Ctrl). In the NORM medium, the amounts on both MAO-treated samples were significantly higher than the amounts in the standard polystyrene cell culture wells (PS) (Fig. 6A). However, after combined cultivation of cells in the NORM and DIF media (7 + 7 days), the amount of type I collagen significantly exceeded the value on PS only in the cells on MA01, but not on the MA01-blasting samples. In accordance with this, the cells cultured on the MA01 samples for 7 + 7 days in the NORM and DIFF media exhibited higher expression of type I collagen than the cells cultured on the MA01-blasting samples, as revealed by the qPCR method (Fig. 6C).

The mineralization of the ECM, measured by the deposition of calcium (Fig. 6B) by the cells, was also evaluated for both types of media, i.e. the NORM medium stimulating cell proliferation and the DIF medium stimulating cell differentiation. Both MAO-modified samples showed similar mineralization values, irrespective of the type of cultivation media. The cells on the MA01 samples after 14 days in the NORM medium showed significantly higher calcium deposition than the Ctrl and PS samples. This more pronounced mineralization, which is also regarded as a marker of osteogenic cell differentiation, corresponds with the described lower proliferation of cells on the MAO-treated samples, as it is known that the cells which are not proliferating tend to differentiate (for a review, see^29,32^). However, this difference among the Ti–6Al–4V-based samples was lost when the cells were cultured in the DIF medium. Under these conditions, the highest calcium deposition was detected in the cells cultured in PS wells. Both MAO-treated samples showed significantly lower mineralization than the PS samples, and slightly, albeit not significantly, decreased mineralization in comparison with the Ctrl samples.

The osteogenic differentiation of the cells cultured on the tested samples was also characterized by qPCR analysis. The expression of three selected markers was investigated: collagen type I (COL1A1 gene), alkaline phosphatase (ALPL gene), and osteocalcin (BGLAP gene). Collagen type I is considered an early marker of cell osteogenic differentiation, as it is secreted by osteoblasts during the deposition of new ECM, and it accounts for almost 90% of the ECM in the bone tissue. Alkaline phosphatase is active during cell mineralization of the surrounding ECM, and in various studies it is considered either as an early marker of osteogenic cell differentiation or as a medium-term marker. Osteocalcin is produced by osteoblasts later during the mineralization phase; it therefore serves as a late marker of osteogenic cell differentiation^40–42^.

The cells grown on the tested samples for 7 days in the NORM medium did not show any significant differences in the expression of COL1A1 gene among the samples, presumably due to the large data spread. From day 7 to day 14 of cultivation in the NORM medium, the expression of COL1A1 in the cells on all samples declined. This can be explained by the fact that collagen type I, being an early osteogenic marker, is downregulated after the cells start differentiating^41^. Nevertheless, after 14 days of cultivation in the NORM medium, the expression values became significantly higher for the cells on the MA01 samples than for the cells on the Ctrl samples (Fig. 6C). After combined cultivation of cells for 7 days in the NORM medium and then for 7 days in the DIF medium, the expression of COL1A1 in the cells on the MA01 samples was comparable with the value on the Ctrl samples, but it was significantly higher than in the cells on the MA01-blasting samples. The relatively high expression of COL1A1 in the cells on the MA01 samples, i.e. in the cells with the lowest proliferation activity (Fig. 5A,C), can be explained by the fact that the less-proliferating cells often synthesize specific molecules and start their differentiation program (for a review, see^29,32^). However, in the cells on the MA01-blasting samples, where the cell proliferation activity was also low, the expression of type I collagen was lower than on the Ctrl samples (Fig. 6C).

Expression data for ALPL gene observed after 7 days of cultivation in the NORM medium showed that the cells on the MA01 and MA01-blasting samples exhibited significantly lower amounts of mRNA for this marker than on the Ctrl and PS samples (Fig. 6C), with the lowest values on the MA01 samples. After 14 days of cultivation in the NORM medium, the expression of ALPL in cells on both the MA01 and the MA01-blasting samples increased relatively, and matched more closely with the expression values for cells on the Ctrl and PS samples. The expression of ALPL in cells after combined cultivation in the NORM and DIF media was also similar on all tested samples, showing relatively balanced values with no statistical significance (Fig. 6C).

Similarly as for COL1A1 expression, there were no statistical differences in BGLAP gene expression between the tested samples with cells cultured for 7 days in the NORM medium. After 14 days of cultivation in the NORM medium, the expression of BGLAP generally increased in cells on all tested samples. This was most apparent on the MA01-blasting and Ctrl samples, but the differences among the tested samples did not reach statistical significance. However, after combined cultivation of cells in the NORM and DIF media, the cells growing on the MA01-blasting samples expressed significantly more BGLAP than on the MA01 samples (Fig. 6C).

Taken together, both MAO-treated Ti–6Al–4V samples were less supportive for cell spreading and proliferation than the control (Ctrl) Ti–6Al–4V samples, but they are relatively supportive for osteogenic cell differentiation. The cells on the MA01 samples displayed (1) significantly higher expression of type I collagen (COL1A1), and (2) significantly higher calcium deposition than on the Ctrl samples after 14 days in the NORM medium, (3) significantly higher immunofluorescence of type I collagen in both the NORM and DIF media than on the PS samples, (4) significantly higher calcium deposition in the NORM media than on the PS samples, and (5) significantly higher expression of type I collagen than on the MA01-blasting samples after combined cultivation in the NORM and DIF media.

The cells on the MA01-blasting samples displayed higher expression of (1) alkaline phosphatase (ALPL; 7 days in the NORM medium) and (2) osteocalcin (BGLAP; combined cultivation in the NORM and DIF media) than the cells on the MA01 samples, and (3) higher immunofluorescence of type I collagen in the NORM medium than the cells on PS. However, the cells on the MA01-blasting samples showed lower expression of type I collagen (DIF medium) and of alkaline phosphatase than on the Ctrl sample. In other words, the MA01-blasting samples showed higher osteogenic differentiation only in three cases, and none of these was observed in comparison with the control Ti–6Al–4V alloy sample. At the same time, the MA01 samples were better for osteogenic differentiation in five cases, and two of these were in comparison with the control Ti–6Al–4V alloy sample. The MAO-blasting samples can therefore be considered less suitable for osteogenic cell differentiation than the MA01 samples. From this point of view, the MA01-blasting samples seem to be more appropriate for temporary applications, e.g. in traumatology, for bone screws, nails, wires, staples or plates, where firm osseointegration (and integration with the surrounding tissues in general) is not desirable. However, the MA01 samples, which exhibited the lowest proliferation of Saos-2 cells, also seem to be suitable for temporary and removable bone implants, because the formation of a sufficient mass of new bone tissue by proliferation of osteoblasts is also a prerequisite for the firm osseointegration of an implant^43^.

## Conclusions

Samples of Ti–6Al–4V alloy, i.e. a material currently used in orthopedic surgery (labelled as Ctrl), were modified by the pulsed micro-arc oxidation (MAO) technique in order to improve the tribological properties of the alloy and to modulate its interaction with osteoblasts. The use of a unipolar power supply with a combination of process parameters (voltage, time) and electrolyte composition (pH > 13) made it possible to create a thin layer with tuned chemical composition on top of the Ctrl samples (labelled as MA01 samples). This layer was highly hydrophilic (water drop contact angle 16°, in comparison with 72° on the Ctrl samples), and also exhibited greater surface roughness. Final surface treatment by blasting (MA01-blasting samples) eliminated the rutile crystalline phase and reduced the surface wettability (contact angle ca. 35°). This can be attributed to thinning of the surface oxide layer and to lowering of the surface roughness by blasting, i.e. mechanical finishing.

The cell–material interactions were studied in vitro with the use of human osteoblast-like Saos-2 cells. On day 1 after seeding, the cells on the MA01 and MA01-blasting samples adhered with a smaller cell–material projected area than on the Ctrl samples. In the following days, the proliferation rate of the cells was lower on both types of MAO-treated samples than on the Ctrl samples. This was more pronounced in the MA01 samples, i.e. in the samples with the highest wettability and roughness. However, the cells on the MA01 samples were more active in osteogenic differentiation and in bone matrix mineralization than the cells on the MA01-blasting samples, although these parameters in both MAO-treated samples were mostly similar to or even lower than on the Ctrl samples. The cells on both MAO-treated samples were highly viable. The technology presented here is therefore suitable for surface modification of temporary traumatological implants, where firm osseointegration is not desirable.

## Materials and methods

### Sample preparation

To obtain the modified surface, the samples of titanium alloy Ti–6Al–4V in the form of discs (diameter 15 mm, thickness 2.6 mm) were mechanically pre-treated by machining and were used to apply MAO.

In order to remove traces of the turning tool and to unify the surface prior to MAO application, the samples were treated by tumbling in an HV 20 centrifugal vibrator (OTEC Präzisionsfinish GmbH) using KF 10 plastic grinding wheels and 1.3 kW engine power for 1.5 h. Subsequently, the samples were polished for 1 h by ZSP 3/5 porcelain bodies.

### Micro-arc oxidation

During the MAO process in an alkaline electrolyte, a complex mechanism involving electro-, thermal- and plasma-chemical reaction takes place^44–46^:

$$ {\text{Cathodic}}\;{\text{reduction:}}\;4{\mathbf{H}}^{ + } + 4{\mathbf{e}}^{ - } \to 2{\mathbf{H}}_{2} $$

$$ \begin{aligned} & {\text{Anodic}}\;{\text{oxidation:}}\;2{\mathbf{H}}_{2} {\mathbf{O}} \to {\mathbf{O}}_{2} + 4{\mathbf{H}}^{ + } + 4{\mathbf{e}}^{ - } \\ & \quad \quad \quad \quad \quad \quad \quad \;\;{\mathbf{Ti}} + {\mathbf{O}}_{2} \to {\mathbf{TiO}}_{2} \\ \end{aligned} $$

High voltage switching power supplies in unipolar or bipolar mode are used to achieve the required breakdown voltage and to form an oxide coating. MAO operating conditions depend on the type of electrolyte that is chosen, and its electrical properties^47,48^, in addition to the parameters of the voltage source.

**Figure 7:**
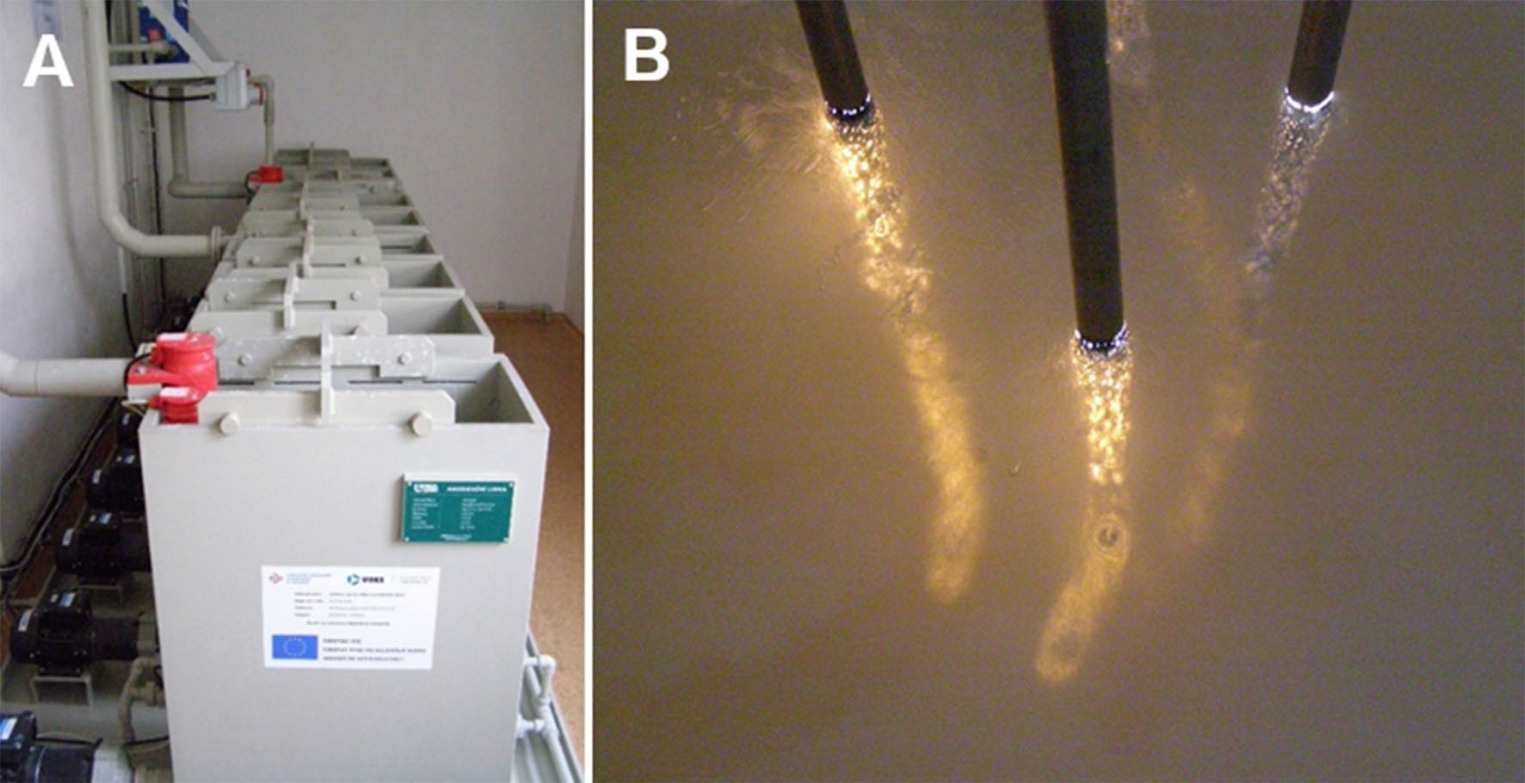
(**A**) MAO pilot plant technology at VUHZ, a.s. (Dobra, Czech Republic). (**B**) Plasma discharge generated on the solid–liquid interface in the electrolyte solution. Author: Simona Gorosova (VUHZ, a.s., Dobra, Czech Republic).

Prior to MAO, the surfaces of the mechanically-treated samples were degreased in an alkaline medium (1 M NaOH for 5 min) and were pickled in a mixture of acids (20 wt.% HNO_3_ and 2 wt.% HF for 1 min). After each of these pretreatment processes, the samples were rinsed 2 times in distilled water for 2 min. The MAO was performed in the pilot plant of VUHZ, a.s. (Dobra, Czech Republic) (Fig. 7). The process was carried out in a strongly alkaline electrolyte (pH > 13, conductivity 38.7 mS cm^−1^) consisting of Na_2_SiO_3_∙9H_2_O and NaOH. A unipolar switching power supply (DEHOR-elspec; Litvinov, s.r.o.) with a voltage of 450 V for 60 min in a highly-cooled bath was used to achieve the necessary plasma conditions for the preparation of the oxide layer. This group of samples was labelled as MA01.

The porous oxide layer after the MAO procedure was removed in the TTB 90 blasting box (GDS Technology, s.r.o.). After a series of tests with organic and ceramic blasting media, the following combination of parameters was determined as the most appropriate: a working distance (15 cm), pressure (1.5 bar), ballotine media (grain size of 65–105 µm). This group of samples was labelled as MA01-blasting.

### Characterization of samples

#### Scanning electron microscopy (SEM), atomic force microscopy (AFM), contact profilometry

The surface morphology and the cross-sections of the MAO-treated samples were observed by field emission scanning electron microscopy (FE-SEM, Quanta 450 FEG, FEI Company, USA). The chemical composition of the MAO-created coating was analyzed using an energy dispersive X-ray spectrometer (EDX, APOLLO X SDD detector, EDAX Inc.). The surface roughness was measured using a contact profilometer device (Talysurf 50, Taylor Hobson). The R_a_, R_z_ and RS_m_ parameters were determined. The R_a_ parameter, i.e. the average roughness, is defined as the arithmetic average of the absolute values of the profile heights along the sampling length. The R_z_ parameter, i.e. the maximum height of the profile, is the absolute vertical distance between the maximum height of the profile peak and the maximum depth of the profile valley depth along the sampling length^39^. The RS_m_ parameter, i.e. the mean spacing of the profile irregularities, is the mean value of the spacing between the profile irregularities within the evaluation length^38^. The topography of the samples was investigated by atomic force microscopy (AFM, NEXT Solver, NT-MDT). The measurements were carried out in contact mode (x, y: 50 × 50 µm).

#### X-ray diffraction (XRD) and X-ray photoelectron spectroscopy (XPS)

The crystalline phases of the MAO-created coating were determined by the X-ray powder diffraction patterns (XRPD) with CoK_α_ irradiation (*λ* = 1.789 Å) and a Bruker D8 Advance diffractometer (Bruker AXS, Germany) equipped with a fast position sensitive VÅNTEC 1 detector. The samples were rotated during the analysis and the measurements were carried out in Lock Coupled mode. The operation conditions of the Co lamp were U = 35 kV, I = 25 mA, and the scanning speeds were 0.03° and 0.8° s^−1^. The ICDD PDF 2 Release 2014 database was used for evaluating the phase composition.

Chemical analyses of the surface of the prepared samples were performed using XPS (X-ray Photoelectron Spectroscopy) in an ultra-high vacuum chamber with a base pressure higher than 2 × 10^−9^ Torr. The spectra were obtained using an Omicron EA-125 electron energy analyser and a dual anode X-ray source. An aluminium Kα_1,2_ line with primary energy 1486.6 eV was used to stimulate the emission of photoelectrons. The films were studied as-prepared, without any additional cleaning.

#### Contact angle and surface energy measurements

The wettability of the Ti–6Al–4V samples was evaluated by the sessile drop technique with the use of two probe liquids (distilled water, glycerol). Eight droplets of 3 μl volume for each liquid were used per sample. The measurements and the subsequent analysis of the static contact angles and the solid surface energies were performed by a Krüss Drop Shape Analyzer 100 machine with Drop Shape Analyzer 4 software (both Krüss GmbH, Germany), using the Owens–Wendt–Rabel–Kaelble method.

#### Tribological testing

The friction and wear testing were done using a pin-on-disc tribometer (CSM Instruments). The tests were performed at room temperature in air and in phosphate-buffered saline (PBS; 137.0 mM NaCl, 2.7 mM KCl and 10 mM phosphate buffer solution in distilled water with pH 7.4 at 25 °C), which was used to simulate the human body environment.

An Al_2_O_3_ ball with a diameter of 6 mm was used as the sliding counterpart. The test parameters were: normal load (1 N), linear sliding speed (50 mm s^−1^), number of laps (5000), radius for air (5 mm) and radius for PBS (6 mm). The surface wear of the Al_2_O_3_ ball was analysed using an Olympus DSX1000 digital microscope, and the geometry of the wear track was measured using a Zygo NewView 72003D optical profilometer.

### Cell culture methods

#### Cell seeding and cultivation

The Ti–6Al–4V samples used in all experiments (MA01, MA01 blasting, MAO-untreated Ctrl) were sterilized in ethanol for 2 h, were inserted into 24-well plates (TPP Techno Plastic Products, Trasadingen, Switzerland) and were seeded with human osteoblast-like Saos-2 cells (ATCC-HTB-85, Chemos GmbH & Co KG, Regenstauf, Germany). The initial seeding density for all experiments was 20,000 cells per well (approx. 11,000 cells/cm^2^), except in the qPCR experiments, where a higher seeding density of 30,000 cells per well (approx. 16,000 cells/cm^2^) was needed in order to increase the RNA yield. As an additional control for all experiments, the cells were seeded into standard polystyrene wells (PS) of 24-well plates.

Two types of cultivation media were used for the experiments. The first type was a normal growth medium (NORM), which contained McCoy 5A cultivation medium (Sigma Aldrich, USA), 15% of Fetal Bovine Serum (FBS; GIBCO, Life Technologies, USA) and gentamicin (40 μg/ml). The second type was a differentiation medium (DIF), which had the same basic composition as NORM, but additionally it was supplemented with β-glycerolphosphate (10 mM), L-glutamine (2 mM), ascorbic acid (50 μg/ml), dihydroxyvitamin D_3_ (10^−6^ M) and dexamethasone (10^−8^ M). According to Jørgensen et al*.*^49^, these media supplements stimulate cell osteogenic differentiation and the production of osteogenic markers in osteoblasts. Dexamethasone, for example, is known to stimulate alkaline phosphatase (ALP) production in cells, and to increase their receptor sensitivity to dihydroxyvitamin D_3 _^50^.

For a proliferation assessment, the cells were cultured for 1, 4, 7 and 14 days in the NORM media at 37 °C and 5% CO_2_ saturation of the air atmosphere. For an assessment of the differentiation, the cells were seeded and cultured for 7 days in the NORM medium, and then the medium was changed to either the NORM medium or the DIF medium, in which the cells were cultivated for an additional 7 days. In other words, the cells were cultivated either for 7 + 7 days in the NORM medium, or for 7 + 7 days in the NORM and DIF medium. The media were changed on every 3rd day of cultivation.

#### Initial cell spreading area, proliferation, viability

In order to evaluate the size of the initial cell spreading areas on day 1 after seeding and the cell population densities on days 1, 4, 7 and 14 of cultivation in the NORM medium, the cells were washed with Phosphate-Buffered Saline, were fixed with 70% frozen ethanol (− 20 °C, 10 min) and were stained with fluorescent dyes for 1 h at room temperature in the dark. Texas Red C_2_ Maleimide (20 ng/ml in PBS; red stain) was used to visualize the cell membrane and the cytoplasmic proteins. Hoechst #33258 (5 μg/ml in PBS; blue stain) was used to visualize the cell nuclei.

On days 1, 4 and 7 of cultivation in the NORM medium, the cells were washed with PBS and were stained with LIVE/DEAD Viability/Cytotoxicity kit (ThermoFisher Scientific, USA) according to the manufacturer’s instructions. Staining with calcein AM (green stain) and with ethidium homodimer 1 (red stain) made it possible to distinguish between live cells (green) and dead cells (red), and to determine the cell numbers and the cell viability in selected time intervals. Additional staining of the cell nuclei with Hoechst #33258 (blue stain) was performed on day 7 for easier cell counting.

The fluorescence signal was viewed and was photographed with an Olympus IX51 epifluorescence microscope (obj. 10×, 20×), equipped with a DP70 camera (both Olympus Corp., Japan). The microphotographs (15 per sample/well) were analyzed in ImageJ FIJI software (https://imagej.net/Fiji^51^;). The cell areas were measured using Altas software (Tescan Ltd., Czech Republic). The initial cell spreading area was presented in μm^2^ as a mean ± S.E.M (Standard Error of Mean). The cell population density is presented as medians with IQR of cell number per cm^2^.

#### Production of collagen type I

The production of collagen type I, which is considered as an early marker of osteogenic cell differentiation, was evaluated at protein level after 7 + 7 days of cultivation in the NORM medium, or after combined cultivation for 7 + 7 days in the NORM and DIF medium. The cells on the tested samples were fixed with frozen 70% ethanol (− 20 °C, 10 min) and were stained by immunofluorescence for collagen type I. A solution of 1% bovine albumin and 0.1% Triton X100 in PBS (20 min, room temperature) was added to the samples in order to block non-specific binding sites for antibodies. After that, the samples were treated with 1% Tween in PBS (20 min) and then with Anti type I Collagen Rabbit primary antibody (1:400 in PBS; Cosmo Bio Co., Ltd., USA, Cat. No. LSL-LB-1197) overnight at 4 °C. Then the secondary antibody Alexa Fluor 488-conjugated F(ab’) 2 fragment of goat anti-rabbit IgG (1:400 in PBS; Molecular Probes, ThermoFisher Scientific, USA, Cat. No. A-11070; green fluorescence) was added for 1 h at room temperature in the dark, along with Hoechst #33258 (5 μg/ml in PBS; blue stain) in order to stain the nuclei. The samples were washed with 1 ml of PBS after each step.

An Olympus IX51 epifluorescence microscope (obj. 20×), equipped with a DP70 camera (both Olympus Corp., Japan), was then used to visualize and to document the fluorescence signal in two separate channels with the same exposition settings for each channel. The intensity of the fluorescence signal of collagen type I was measured in microphotographs (10 per sample/well) using a Fluorescent Image Analyser (Matejka, software^52^). The same single color plane threshold was set for all images to eliminate the non-protein area of the image data. The cumulative sum of all pixel intensities was then evaluated with subtraction of the background fluorescence intensity of the negative staining control. The obtained data were normalized to cell counts for each image separately, and are presented as medians with IQR.

#### Gene expression of osteogenic markers

The osteogenic cell differentiation was also analyzed at mRNA level using Real-Time quantitative PCR (qPCR) in order to evaluate the expression of the following selected genes of interest: collagen type I (COL1A1 gene), an early marker, alkaline phosphatase (ALPL gene), an intermediate marker, and osteocalcin (BGLAP gene), a late marker of osteogenic cell differentiation. Glyceraldehyde 3-phosphate dehydrogenase (GAPDH) was used as a housekeeping (i.e. reference) gene. The cells for these experiments were cultivated for 7 or 14 days in the NORM medium, or consecutively for 7 + 7 days in the NORM and DIF medium to stimulate osteogenic cell differentiation, as previously described.

The Total RNA Purification Plus Micro Kit (Norgen Biotek, Canada) was used according to the manufacturer’s instructions for RNA extraction from the cultured cells. Reverse transcription of RNA (300 ng/µl) to cDNA was performed using ProtoScript First Strand cDNA Synthesis Kit (New England BioLabs, USA) with oligo-dT primers. The reaction ran in a T-Personal Thermocycler (Biometra, Germany). The relative mRNA expression was quantified using SYBR Green (FastStart Universal SYBR Green Master; Roche Diagnostics GmbH, Germany) and Generi Biotech (Czech Republic) primers, the sequences of which are described in Table 3. The iCycler iQTM 5 Multicolor Real Time PCR detection system (BioRad, USA) was used for cDNA amplification in a total reaction volume of 20 μl and in the following cycling conditions: 95 °C (10 min), 40 cycles of 95 °C (15 s) and 60 °C (1 min). The assay was conducted in triplicate. The relative mRNA expression was quantified by the 2^−ΔΔCt^ method. Changes in the expression of the genes of interest were calculated according to the equation:

$$ \Delta \Delta C_{t} = \left( {C_{t}^{target} - C_{t}^{GAPDH} } \right)_{sample} - \left( {C_{t}^{target} - C_{t}^{GAPDH} } \right)_{calibrator} $$

**Table 3:** The primers used for qPCR amplifications are the same as were used in^53^.

Gene	Primer sequence	Product Length [bp]
Collagen type I (COL1A1)	Forward: 5′-CAGCCGCTTCACCTACAGC-3′	83
	Reverse: 5′-TTTTGTATTCAATCACTGTCTTGCC-3′	
Alkaline Phosphatase (ALPL)	Forward: 5′-GACCCTTGACCCCCACAAT-3′	68
	Reverse: 5′-GCTCGTACTGCATGTCCCCT-3′	
Osteocalcin (BGLAP)	Forward: 5′-GAAGCCCAGCGGTGCA-3'	70
	Reverse: 5′-CACTACCTCGCTGCCCTCC-3'	
GAPDH	Forward: 5′-TGCACCACCAACTGCTTAGC-3′	87
	Reverse: 5′-GGCATGGACTGTGGTCATGAG-3′	

The data were normalized according to the gene expression in the cells grown on the PS samples in the NORM medium for 7 days after seeding (PS_7d/NORM, calibrator).

#### Extracellular matrix mineralization

The extracellular matrix (ECM) mineralization was evaluated using the Calcium Colorimetric Assay kit (BioVision, Inc., USA, Cat. No. K380-250) according to the manufacturer’s instructions. The cells were cultivated either for 7 + 7 days in the NORM medium, or consecutively for 7 + 7 days in the NORM and DIF medium. Prior to the assay, all samples of Ti–6Al–4V were moved into a fresh 24-well plate in order to eliminate the influence of the cells growing on the original well bottoms under and around the samples. All samples and PS controls were washed twice with PBS and were left to dry out under non-sterile conditions at room temperature for 1 h. Afterwards, 700 μl of 0.5 M HCl was added to each sample for overnight incubation at 4 °C on an orbital SSL1 shaker. The cells were then scratched with a cell scraper and were collected into clean Eppendorf tubes. Twenty-five μl of solution from each sample were pipetted in triplicate into a 96-well plate (TPP, Techno Plastic Products, Trasadingen, Switzerland), along with 45 μl of Chromogenic Reagent and 30 μl of Calcium Assay Buffer (5–10 min at room temperature in the dark). Immediately after the reaction, the Synergy HT Multi mode Reader (BioTek, USA) was used to measure the absorbance of each well at 575 nm. A calibration curve was created from standards containing known concentrations of calcium so that the absorbance could be converted to the calcium concentration. The data were normalized according to the sample surface area (the well diameter of the polystyrene 24-well plate was 15.4 mm, the sample diameter was 12 mm).

### Statistical analysis

SigmaStat 3.5 (Systat Software Inc., USA) was used for the statistical analysis. The quantitative data were analyzed using Kruskal Wallis One Way ANOVA on Ranks with post hoc Dunn’s analysis and statistical significance at *p* ≤ 0.05, except for qPCR, where the data were analyzed in the form of ΔCt using the One Way ANOVA, Holm–Sidak test, statistical significance at *p* ≤ 0.001. The data are presented either as bar charts with mean ± S.E.M (Standard Error of Mean) or as box plots with median, quartiles and interquartile range, with the exception of qPCR. The data from qPCR are presented as the mean ± S.D. (Standard Deviation) from 3 measurements. All plots were created in GraphPad Prism 8.3.0 (GraphPad Software, USA).
